# Metabolic syndrome and abdominal fat are associated with inflammation, but not with clinical outcomes, in peritoneal dialysis patients

**DOI:** 10.1186/1475-2840-12-86

**Published:** 2013-06-08

**Authors:** Jenq-Wen Huang, Chung-Yi Yang, Hon-Yen Wu, Kao-Lang Liu, Chi-Ting Su, Cho-Kai Wu, Jen-Kuang Lee, Chih-Kang Chiang, Hui-Teng Cheng, Yu-Chung Lien, Kuan-Yu Hung

**Affiliations:** 1 Department of Internal Medicine, National Taiwan University College of Medicine and Hospital, No. 7, Chung-Shan South Road, Taipei 100, Taiwan; 2 Medical Imaging, National Taiwan University College of Medicine and Hospital, Taipei, Taiwan; 3 Integrated Diagnostics and Therapeutics, National Taiwan University College of Medicine and Hospital, Taipei, Taiwan; 4 Department of Internal Medicine, Far Eastern Memorial Hospital, New Taipei City, Taiwan; 5 National Taiwan University College of Medicine and Hospital, Yun-Lin Branch, Yun-Lin County, Taiwan; 6 National Taiwan University College of Medicine and Hospital, Hsin-Chu Branch, Hsin Chu City, Taiwan; 7 Department of Internal Medicine, Buddhist Tzu Chi General Hospital, Taipei Branch, New Taipei City, Taiwan; 8 Cardiovascular Center & Department of Clinical Pathology, Far Eastern Memorial Hospital, New Taipei City, Taiwan

**Keywords:** Abdominal fat, Atherogenic indices, Beta blocker, Renin-angiotensin system blocker, Computed tomography, Metabolic syndrome, Peritoneal dialysis

## Abstract

**Background:**

In the general population, metabolic syndrome (MetS) is correlated with visceral fat and a risk factor for cardiovascular disease (CVD); however, little is known about the significance of abdominal fat and its association with inflammation and medication use in peritoneal dialysis (PD) patients. We investigated the relationship of visceral fat area (VFA) with C-reactive protein (CRP) levels and medication use in PD patients and followed their clinical outcomes.

**Methods:**

In a prospective study from February 2009 to February 2012, we assessed diabetes mellitus (DM) status, clinical and PD-associated characteristics, medication use, CRP levels, components of MetS, and VFA in 183 PD patients. These patients were categorized into 3 groups based on MetS and DM status: non-MetS (group 1, n = 73), MetS (group 2, n = 65), and DM (group 3, n = 45). VFA was evaluated by computed tomography (CT) and corrected for body mass index (BMI).

**Results:**

Patients in group 1 had smaller VFAs than patients in groups 2 and 3 (3.2 ± 1.8, 4.6 ± 1.9, and 4.9 ± 2.0 cm^2^/[kg/m^2^], respectively*, P* < 0.05) and lower CRP levels (0.97 ± 2.31, 1.27 ± 2.57, and 1.11 ± 1.35 mg/dL, respectively, *P* < 0.05). VFA increased with the number of criteria met for MetS. After adjusting for age, body weight, and sex, CRP and albumin levels functioned as independent positive predictors of VFA; on other hand, the use of renin-angiotensin system blockers was inversely correlated with VFA in PD patients without DM. In the survival analysis, DM patients (group 3) had the poorest survival among the 3 groups, but no significant differences were found between groups 1 and 2.

**Conclusion:**

This study showed that VFA and MetS are associated with CRP levels but cannot predict survival in PD patients without DM. The complex relationship of nutritional parameters to VFA and MetS may explain these results. The type of antihypertensive medication used was also associated with the VFA. The mechanisms behind these findings warrant further investigation.

## Introduction

Visceral fat area (VFA) is known to be correlated strongly with metabolic syndrome (MetS). In the general population, increased VFA is an independent risk factor for atherosclerosis [1-4]. Because VFA plays a key role in the development of diabetogenic, atherogenic, prothrombotic, and proinflammatory MetS, VFA reduction has been proposed as a strategy to prevent atherosclerosis in MetS patients [5].

In patients requiring dialysis, disturbances in lipid and carbohydrate metabolism, which are common, have been associated with VFA and may develop into MetS. In patients with chronic kidney disease (CKD), a higher body mass index (BMI) has been reported to be inversely correlated with mortality; however, other studies have suggested that body composition is a more accurate predictor of survival than BMI [6,7].

VFA can be evaluated accurately by computed tomography (CT) at the level of the umbilicus. In hemodialysis (HD) patients, VFA was found to be the best predictor of atherogenic index (AI), triglyceride (TG) level, and degree of atherosclerosis after correcting for BMI [8,9]. Inflammation is another important component of MetS; in HD [10] and peritoneal dialysis (PD) patients [11], VFA has been found to be associated with increased levels of inflammatory markers such as C-reactive protein (CRP).

An increased glucose concentration in peritoneal fluid results in increased carbohydrate absorption by the peritoneal membrane. The average concentration of glucose in the peritoneal fluid of a PD patient predicts survival and is associated with higher serum glucose levels [12-14]. In one study, approximately one-third of PD patients without DM exhibited insulin resistance [15]; other studies have shown a high incidence (≥50%) of MetS in this population [16,17]. In PD patients, both insulin resistance and high glucose absorption lead to increased VFA, and thus are associated with higher adipokine concentrations than those in individuals with normal renal function [18]. Therefore, the significant contribution of VFA to increased BMI and the association of VFA with CRP make the elucidation of its role in PD patient outcomes complex.

Some drugs may affect glucose and lipid metabolism and, therefore, influence abdominal fat. Renin-angiotensin system (RAS) blockers have been reported to reduce VFA and decrease vascular inflammation [19,20]. Traditional beta blockers can reduce insulin sensitivity and increase the risk of DM and dyslipidemia [21]. Statins are also used to manage dyslipidemia and insulin resistance [22]. PD patients commonly use these medications to treat hypertension, heart disease, or dyslipidemia; however, the effects of these medications on VFA have never been studied.

This study aimed at clarifying the associations among VFA, MetS, inflammation, and medication use and their corresponding effects on PD patient outcomes. We found that medication use, inflammation, and MetS were all associated with VFA in the PD population.

## Materials and methods

### Study design

In February 2009, patients aged >20 years who had undergone maintenance PD for more than 3 months were enrolled. Pregnant women and patients who had undergone CT scanning during the previous 6 months were excluded. After providing informed consent, each patient underwent a non-contrast abdominal CT, and a blood sample was obtained to measure biomarkers. Patients fasted for 6 hours prior to phlebotomy. The blood samples were immediately centrifuged at 3,000 rpm and 4°C, and the resulting plasma samples were frozen at −80°C until analysis.

#### Ethical considerations

This study was approved by the ethics committee of this hospital in documents NTUH-REC No. 200808062R and NTUH-REC No. 201104032RC. As stated, patients provided written informed consent before they entered the study.

### Assessment of abdominal fat by computed tomography

The imaging of each subject was performed on a 64-MDCT scanner (LightSpeed VCT; GE Healthcare, Milwaukee, WI), and the umbilicus cut was analyzed for VFA [23]. Image analysis software (ImageJ, version 1.45; National Institutes of Health, Bethesda, MD) was used at an attenuation range of −50 to −250 Hounsfield units to quantify abdominal adipose tissue areas in cm^2^. The subcutaneous fat area (SFA) was clearly visible and defined as the extraperitoneal fat between skin and muscle. Intra-abdominal tissue at the same density as SFA was defined as VFA. The total fat area (TFA) was the sum of SFA and VFA. The images were reviewed by radiologists who were blinded to the clinical characteristics of the PD patients. The 3 indicators of fat area were corrected for BMI (cm^2^/[kg/m^2^]) because fat area relates to body mass [8].

### Clinical characteristics and follow-up

Clinical and dialysis data of PD patients were recorded and included medication (RAS blockers, beta blockers, or statin), results of PD-associated peritoneal equilibration test (PET), dialysis Kt/V, residual renal function, and normalized protein catabolic rate (nPCR). Routine biochemical studies were also recorded and included urea nitrogen, creatinine, albumin, hemoglobin, CRP, lipid profile, total cholesterol (CHO), triglyceride (TG), high-density lipoprotein (HDL), and low-density lipoprotein (LDL).

Two atherogenic indices (AIs) were derived from the lipid profile, according to the following equations: AI1 = log (TG/HDL) [24] and AI2 = non-HDL cholesterol/HDL = (T-CHO-HDL)/HDL [8].

The dialysate prescribed to each PD patient during 2009 was also recorded and used to calculate the glucose load in these patients, as previously described [12-14]. Glucose load 1 represents the average glucose concentration in the total PD fluid, including extraneal and nutrineal. Glucose load 2 represents the average glucose concentration of the glucose-containing dialysate only.

After they had undergone an abdominal CT, patients were monitored for the following events: hospitalization, peritonitis, technique failure, cardiovascular disease (CVD), and mortality. Cardiac disease, cerebrovascular disease, and severe ischemic events were categorized as CVD. Patients who received transplants were censored in technique survival and mortality. Transplant admissions were not considered episodes of hospitalization. Follow-up was continued until February 2012.

### Metabolic syndrome

MetS was diagnosed according to the definition in the National Cholesterol Education Program Adult Treatment Panel III [25], which requires fulfillment of at least 3 of the following criteria: abnormal waist circumference, TG >150 mg/dL, HDL < 40 mg/dL in men or < 50 mg/dL in women, blood pressure >130/85 mmHg or active treatment with antihypertensive agents, and fasting blood glucose >100 mg/dL. Because waist measurements are inaccurate in PD patients, we substituted BMI >25 kg/m^2^ for this value, as has been done in other studies [17].

### Statistical analysis

All continuous variables are reported as mean ± SD (with 95% confidence intervals as appropriate), and all categorical variables are reported as frequencies or percentages. Comparison between groups was done using the student *t*-test, non-parametric test, or one-way analysis of variance (ANOVA). The differences in frequency were tested by *χ*^2^ analysis. The relationships between variables were tested by Pearson’s correlation. The independent determinants of a variable were determined by multiple linear regression analysis. The adjusted variables were stated for each analysis. Peritonitis incidence in PD patients was compared by Poisson analysis. Kaplan-Meier survival analysis was used to compare survival between groups. *P* values < 0.05 were considered significant. Statistical analyses were conducted using SPSS 13.0 for Windows (SPSS Inc., IL USA).

## Results

### Clinical and PD-associated parameters among patients

A total of 183 PD patients were enrolled in this study. There were 146 patients without DM who were further classified as non-MetS (n = 73, group 1) or MetS (n = 65, group 2). The remaining patients were classified as DM (n = 45, group 3), including 5 patients met the criteria for DM after they had begun PD. Besides differing in the criteria met for MetS, patients in group 2 also had higher levels of albumin and CRP, higher AI1 and AI2, more abdominal fat indicators, and lower levels of D/P creatinine and nPCR than patients in group 1 (Table 1). DM patients (group 3) had more significant histories of coronary artery disease (CAD), older ages, higher BMIs, higher glucose loads, increased AI1 and AI2, and more abdominal fat than patients in group 1. The PD duration in the DM patients was the shortest and the nPCR was the lowest of the 3 groups. These results indicate that MetS patients are similar to DM patients in that they have higher AIs and CRP levels, and more abdominal fat, but MetS patients have less significant CAD histories.

**Table 1:** Clinical characteristics and biochemical parameters among peritoneal dialysis(PD) patients without metabolic syndrome (MetS, group 1), PD patients with MetS (group 2), and diabetes mellitus (DM) patients (group 3)

	**1**			**2**			**3**		
	**Non-MetS, n = 73**			**MetS, n = 65**			**DM, n = 45**		
	**Mean**	**±**	**SD**	**Mean**	**±**	**SD**	**Mean**	**±**	**SD**
Women	39			40			19		
CAD	6			8			17^*^		
Hypertension	55			58^*^			40		
RAS blocker	37			34			19		
Beta blocker	35			49^*^			28		
Statin	17			24			18		
Age	51	±	14	53	±	12	58	±	11^*^
PD vintage (months)	46	±	45	49	±	36	22	±	19^*^
Body weight (kg)	57	±	9	61	±	12^*^	68	±	13^*^
Renal KT/V	0.22	±	0.33	0.17	±	0.32	0.18	±	0.23
Peritoneal KT/V	1.87	±	0.41	1.92	±	0.35	1.79	±	0.31
4-h D/P Cre	0.67	±	0.10	0.64	±	0.09^*^	0.68	±	0.11
D4/D0 Glu	0.37	±	0.07	0.40	±	0.06^*^	0.39	±	0.07
Glucose exposure (kg/year)	53	±	19	56	±	20	58	±	22
Dialysate glucose load (g/dL)	1.86	±	0.33	1.87	±	0.31	2.11	±	0.31
nPCR (gm/[kg/day])	1.02	±	0.19	0.93	±	0.16^*^	0.90	±	0.21^*^
Albumin (g/mdL)	3.9	±	0.3	4.1	±	0.3^*^	4.0	±	0.4
Hemoglobin (g/dL)	10.2	±	1.2	10.0	±	1.6	10.2	±	1.0
Creatinine (mg/dL)	11.5	±	2.9	11.2	±	2.7	10.9	±	2.6
Glucose (mg/dL)	92	±	11	106	±	24^*^	139	±	52^*^
Cholesterol (mg/dL)	197	±	44	202	±	48	186	±	41
Triglyceride (mg/dL)	116	±	54	273	±	208^*^	203	±	133^*^
HDL (mg/dL)	48	±	15	37	±	7^*^	36	±	12^*^
LDL (mg/dL)	100	±	39	87	±	42	85	±	37^*^
AI1	0.36	±	0.25	0.80	±	0.26^*^	0.67	±	0.35^*^
AI2	3.31	±	1.31	4.58	±	1.31^*^	4.42	±	1.58^*^
CRP (mg/dL)	0.97	±	2.31	1.27	±	2.57^a^	1.11	±	1.35^a^
Cardiothoracic ratio (%)	48	±	7	50	±	7	53	±	6
SFA (cm^2^/[kg/m^2^])	4.8	±	2.4	6.1	±	2.0^*^	7.3	±	2.8^*^
TFA (cm^2^/[kg/m^2^])	8.0	±	3.6	10.7	±	3.4^*^	12.1	±	3.8^*^
VFA (cm^2^/[kg/m^2^])	3.2	±	1.8	4.6	±	1.9^*^	4.9	±	2.0^*^

^*^P < 0.05 for comparison to group 1 patients by the student *t*-test.

^*a*^*P* < 0.05 for comparison to group 1 patients by the non-parametric *t*-test.

*SD* standard deviation, *AI1* log (TG/HDL); *AI2* non-HDL cholesterol/HDL, *BMI* Body mass index, *CAD* coronary artery disease, *CRP* C-reactive protein, *HDL* high density lipoprotein, *LDL* low density lipoprotein, *RAS* renin-angiotensin system, *SFA* subcutaneous fat area, *TFA* total fat area, *VFA* visceral fat area.

### Predictors for VFA, SFA, and TFA

**Figure 1:**
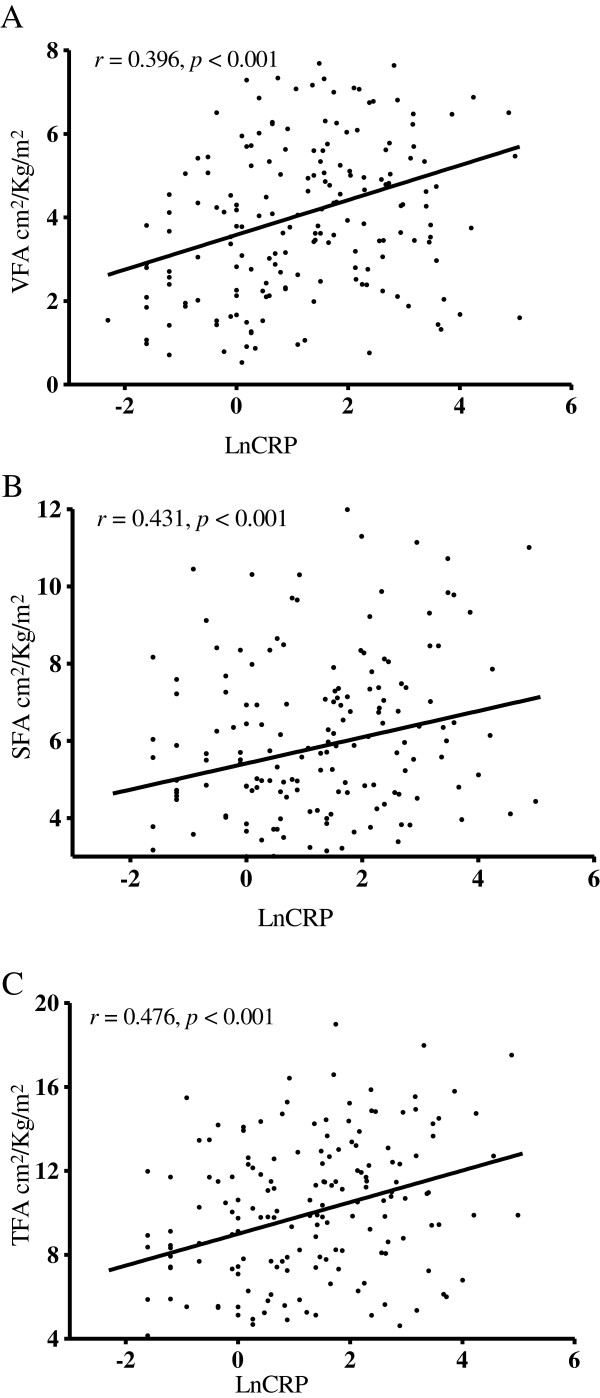
**The relationship between fat area and CRP was analyzed with Pearson correlation.** Different components of abdominal fat area were all positively correlated with CRP.

Because DM significantly influences lipid and carbohydrate metabolism and fat distribution, we analyzed the correlation between VFA and other variables in the groups. Because CRP was not evenly distributed, we log-transformed the CRP data for analysis. We applied the Pearson’s correlation to define the relationship between CRP levels and fat components. In non-DM patients, CRP levels had a significant positive correlation with VFA (r = 0.396*, P* < 0.001; Figure 1A), SFA (r = 0.431*, P* < 0.001; Figure 1B), and TFA (r = 0.476*, P* < 0.001; Figure 1C). Otherwise, fat area correlated proportionally with age, body weight (BW), CHO, TG, AIs, and fasting glucose (Table 2). These abdominal fat parameters were inversely correlated with peritoneal KT/V, creatinine, and nPCR. The cardiothoracic ratio, which is inversely related to heart function, also had a positive correlation with abdominal fat. The use of RAS blockers had a negative correlation with abdominal fat, whereas the use of beta blockers had a positive one (Table 2). In general, the correlations were similar in DM patients, except that CRP level and type of antihypertensive medication had no correlations with abdominal fat.

**Table 2:** Correlations between abdominal fat and clinical parameters in non-DM (n = 138) and DM (n = 45) PD patients

	**Non-DM, n = 138**						**DM, n = 45**					
	** VFA**		** SFA**		** TFA**		** VFA**		** SFA**		** TFA**	
Sex	−0.04		0.31	^*^	0.17	^*^	0.02		0.23		0.18	
CAD	0.06		0.01		0.04		0.25		0.02		0.15	
Hypertension	−0.15		0.06		−0.04		0.31	^*^	0.06		0.20	
RAS blocker	−0.17	^*^	−0.17	^*^	−0.20	^*^	−0.06		−0.13		−0.13	
Beta blocker	0.15	^*^	0.27	^*^	0.24	^*^	0.08		−0.12		−0.04	
Statin	0.10		0.14		0.14		−0.08		−0.12		−0.13	
Age	0.40	^*^	0.35	^*^	0.43	^*^	0.50	^*^	0.06		0.31	^*^
PD vintage	0.00		−0.09		−0.06		−0.20		−0.04		−0.13	
Body weight	0.38	^*^	0.15		0.29	^*^	−0.08		0.37	^*^	0.23	
BMI	0.44	^*^	0.41	^*^	0.49	^*^	0.02		0.54	^*^	0.41	^*^
Renal KT/V	0.07		0.15		0.13		0.14		0.05		0.10	
Peritoneal KT/V	−0.18	^*^	−0.14		−0.18	^*^	−0.12		0.10		0.02	
4 hr D/P Cre	−0.05		−0.04		−0.05		−0.02		0.19		0.13	
D4/D0 Glu	0.15		0.10		0.14		−0.03		−0.15		−0.13	
Glucose exposure	−0.07		−0.07		−0.08		0.01		0.22		0.16	
Dialysate glucose load	−0.02		0.02		0.00		0.09		−0.07		0.00	
nPCR	−0.23	^*^	−0.27	^*^	−0.28	^*^	−0.25		−0.21		−0.28	
Albumin	0.22	^*^	0.06		0.15		0.09		−0.03		0.02	
Hemoglobin	0.05		0.03		0.05		0.01		−0.37	^*^	−0.26	
Creatinine	−0.13		−0.29	^*^	−0.25	^*^	−0.35	^*^	−0.20		−0.32	^*^
Glucose	0.17	^*^	0.10		0.15		0.11		0.29	^*^	0.27	
Cholesterol	0.16		0.24	^*^	0.23	^*^	0.24		0.22		0.28	
Triglyceride	0.27	^*^	0.12		0.22	^*^	0.54	^*^	0.34	^*^	0.53	^*^
HDL	−0.27	^*^	0.01		−0.14		−0.26		−0.22		−0.30	^*^
LDL	−0.02		0.06		0.03		−0.11		0.09		0.01	
AI1	0.42	^*^	0.19	^*^	0.34	^*^	0.55	^*^	0.41	^*^	0.59	^*^
AI2	0.39	^*^	0.18	^*^	0.32	^*^	0.45	^*^	0.31	^*^	0.46	^*^
LnCRP	0.36	^*^	0.16		0.29	^*^	0.02		0.19		0.15	
Cardiothoracic ratio (%)	0.25	^*^	0.32	^*^	0.33	^*^	0.32	^*^	0.23		0.33	^*^

Abdominal fat was categorized as visceral fat area (VFA), subcutaneous fat area (SFA), and total fat area (TFA), and all were corrected for body mass index (BMI).

^*^P < 0.05 with Pearson’s correlation.

In addition, we used multiple linear regression analysis to determine independent predictors, after adjusting for age, sex, and BW. In non-DM patients, creatinine associated negatively with all fat area indicators (Table 3A). CRP levels were associated positively with VFA and TFA. The use of RAS blockers negatively predicted VFA, whereas the use of beta blockers associated positively with SFA. Both types of medications correlated with TFA. In DM patients, the fat area was associated only with age, sex, and lipid parameters (Table 3B).

**Table 3:** Independent determinants of abdominal fat area by multiple linear regression analysis and adjusted for age and sex in non-DM (A) and DM (B) PD patients

**A.**												
	**VFA**				**SFA**				**TFA**			
	**B**	**±**	**SE**	***P***	**B**	**±**	**SE**	***P***	**B**	**±**	**SE**	***P***
Constant	−7.58	±	1.89	< 0.001	−6.86	±	1.77	< 0.001	−13.34	±	2.86	< 0.001
Woman	0.51	±	0.33	0.13	2.64	±	0.40	< 0.001	2.81	±	0.62	< 0.001
Age (per 10 years)	0.40	±	0.10	< 0.001	0.43	±	0.12	< 0.001	0.76	±	0.19	< 0.001
BW (per 10 kg)	0.95	±	0.15	< 0.001	1.26	±	0.18	< 0.001	2.20	±	0.27	< 0.001
Creatinine	−0.17	±	0.05	< 0.01	−0.19	±	0.06	< 0.01	−0.37	±	0.09	< 0.001
Albumin	0.95	±	0.37	< 0.05								
AI2	0.30	±	0.09	< 0.001								
LnCRP	0.27	±	0.08	< 0.001					0.43	±	0.14	< 0.01
RAS blocker	−0.62	±	0.25	< 0.05					−0.92	±	0.47	0.05
Beta blocker					1.03	±	0.30	< 0.001	1.35	±	0.45	< 0.01
Cholesterol (per 10 mg/dL)									0.11	±	0.05	< 0.05
D4/D0 Glu (per 0.1)									0.67	±	0.33	< 0.05
R^2^	0.559				0.476				0.600			
**B.**												
	**VFA**				**SFA**				**TFA**			
	**B**	**±**	**SE**	**P**	**B**	**±**	**SE**	**P**	**B**	**±**	**SE**	**P**
Constant	−0.29	±	1.30	0.83	−8.10	±	3.56	< 0.05	−7.60	±	4.78	0.12
Woman	−0.26	±	0.47	0.59	2.75	±	0.73	< 0.001	2.57	±	0.97	< 0.05
Age (per 10 years)	0.71	±	0.21	< 0.01	0.21	±	0.33	0.53	0.80	±	0.44	0.08
Cholesterol (per 10 mg/dL)	0.07	±	0.02	< 0.001								
BW (per 10 kg)					1.28	±	0.31	< 0.001	1.20	±	0.41	< 0.01
AI1					2.38	±	1.02	< 0.05	4.93	±	1.37	< 0.001
R^2^	0.445				0.464				0.491			

### Abdominal fat area in MetS

**Figure 2:**
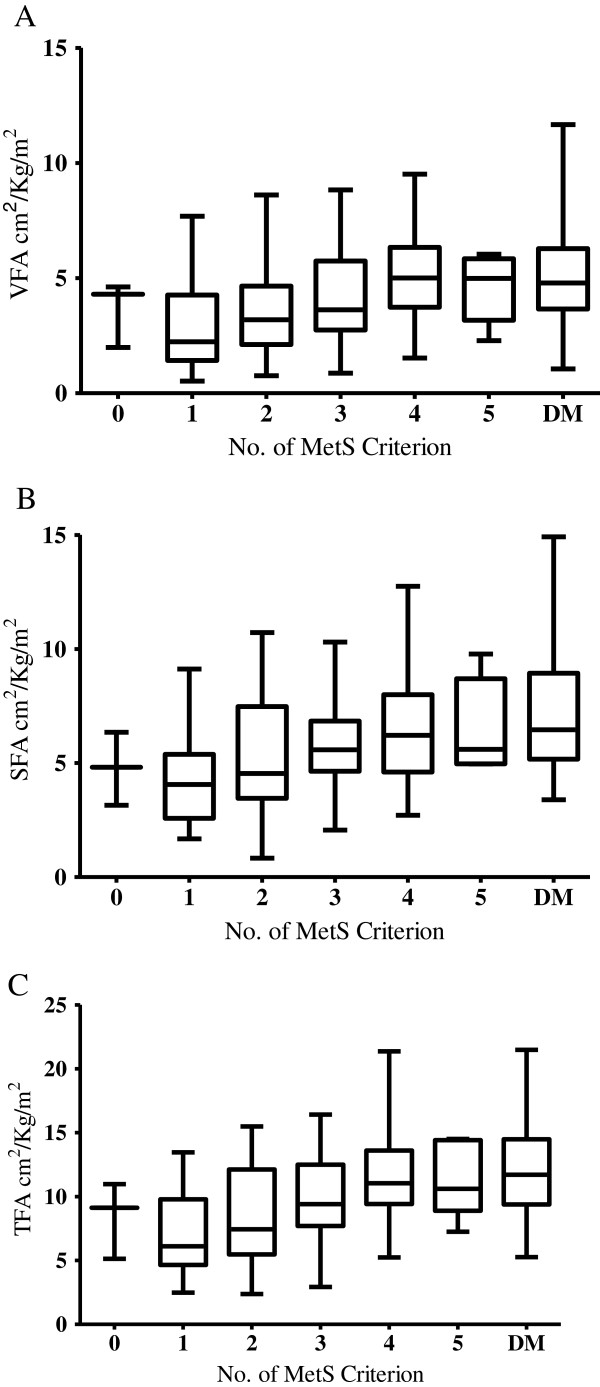
**The fat area increased as the increment of number of metS criterion in each component of fat area (A) VFA, (B) SFA, and (C) TFA (p < 0.001 with ANOVA).** The DM patients was categorized as another group in the last bar.

Since fat area correlated with lipid and glucose metabolism, both of which reflect metabolic dysfunction, we analyzed the fat area in all PD patients without DM and categorized the patients into 6 groups based on the number of criteria they met for MetS. Four patients did not meet any criteria of MetS, 35 fulfilled 1 criterion, 39 fulfilled 2 criteria, 45 fulfilled 3 criteria, 44 fulfilled 4 criteria, and 16 fulfilled all 5 MetS criteria. VFA, SFA, and TFA increased as the number of criteria met increased (Figure 2; *P* < 0.001 by ANOVA).

### Effects of MetS on PD patient outcomes

PD patients without DM were analyzed further for each criterion of MetS fulfilled by comparing VFA, CRP, PD dialysate glucose load, annual glucose exposure, CAD history, and CVD comorbidity. CRP levels were higher in patients who fulfilled any single criterion except for hypertension (Table 4). PD glucose load and exposure were similar for each criterion. Finally, none of the criteria for MetS resulted in significant differences in heart size, CAD history, or follow-up CVD events.

**Table 4:** Comparison of glucose exposure, inflammation, and CV comorbidity among non-DM PD patients categorized according to each criterion of metabolic syndrome

	**Triglyceride**		**High density lipoprotein**		**Hypertension**		**Body mass index**		**Glucose**	
	**−**	**+**	**−**	**+**	**−**	**+**	**−**	**+**	**−**	**+**
	**69**	**69**	**52**	**86**	**22**	**116**	**113**	**25**	**94**	**44**
CRP (mg/dL)	1.0 ± 2.6	1.2 ± 2.3	1.0 ± 2.4	1.2 ± 2.5^*^	2.0 ± 3.7	1.0 ± 2.1^*^	0.9 ± 2.0	2.0 ± 3.7^*^	1.0 ± 2.1	1.4 ± 3.0^*^
VFA (cm^2^/[kg/m^2^])	3.1 ± 1.8	4.6 ± 1.9^*^	3.2 ± 1.8	4.2 ± 2.0^*^	4.6 ± 2.0	3.7 ± 2.0	3.5 ± 1.8	5.4 ± 1.9^*^	3.6 ± 2.1	4.4 ± 1.8^*^
Cardiothoracic ratio (%)	48 ± 6	49 ± 7	48 ± 6	49 ± 7	47 ± 7	49 ± 7	48 ± 7	50 ± 5	48 ± 7	50 ± 7
Albumin (g/dL)	3.9 ± 0.3	4.1 ± 0.3^*^	3.9 ± 0.4	4.1 ± 0.3^*^	4.0 ± 0.3	4.0 ± 0.4	4.0 ± 0.3	4.1 ± 0.4	4.0 ± 0.3	4.1 ± 0.4
Renal KT/V	0.21 ± 0.32	0.19 ± 0.32	0.30 ± 0.37	0.14 ± 0.28^*^	0.10 ± 0.16	0.22 ± 0.34^*^	0.17 ± 0.29	0.32 ± 0.44^*^	0.20 ± 0.32	0.21 ± 0.32
Glucose load (g/dL)	1.88 ± 0.33	1.86 ± 0.31	1.80 ± 0.29	1.91 ± 0.33	1.82 ± 0.31	1.88 ± 0.32	1.86 ± 0.32	1.89 ± 0.34	1.87 ± 0.33	1.85 ± 0.29
Glucose exposure (kg/year)	56 ± 20	53 ± 19	49 ± 19	58 ± 20^*^	53 ± 12	55 ± 21	54 ± 20	57 ± 21	55 ± 20	53 ± 19
CAD History	7	7	3	11	3	11	12	2	8	6
CVD Event	5	5	3	7	1	9	8	2	7	3

*P < 0.05 compared by student *t*-test, non-parametric *t*-test, or Chi-test.

*CAD* coronary artery disease, *CRP* C-reactive protein, *CVD* cardiovascular disease.

The Kaplan-Meier survival analysis was used to compare survival among non-MetS, MetS, and DM patients. Patients with DM had the shortest survival, but MetS did not have an effect on survival outcome (Figure 2).

## Discussion

In this 3-year prospective study, we have demonstrated that VFA, SFA, and TFA increased with the number of MetS criteria met in PD patients. VFA and MetS were also associated with inflammation in PD patients without DM. The use of both RAS blockers and beta blockers was associated with the area of abdominal fat. However, neither MetS nor abdominal fat was associated with technique failure, hospitalization, CVD events, or mortality.

It has been reported that renal failure, especially in association with DM, is associated with the occurrence of CVD events and is a predictor of poor prognosis in patients with acute myocardial infarction [26,27]. MetS is similar to DM in insulin resistance and abnormal glucose and lipid metabolism, and MetS was diagnosed in nearly half of the non-DM PD patients in this study and has been observed in a higher percentage of patients in other studies [16,17]. This result is not surprising because one-third of PD patients without DM are glucose intolerant [15]. In addition, PD elevated the daily dialysate glucose load by 1.86 g/dL and the annual glucose exposure by 53–56 kg (Table 1), which may affect patients with low HDL levels (Table 4). As in the generalized population [28], PD patients showed an association between MetS and abdominal fat.

Although waist circumference had been reported to be a reliable marker of abdominal adiposity in PD patients [29], waist circumference may be distorted by PD fluid instillation. Fat area measured by the CT scan, as in the present study, would appear to be the more accurate method of measurement. Abdominal fat area increased with the number of criteria fulfilled in PD patients (Figure 2).

MetS in PD patients was also related to inflammation, which is the primary cause of obesity-linked insulin resistance and not only a mere consequence of obesity [30]. Fat accumulation has been linked to inflammation that is characterized by increased CRP levels [31], as shown in the present study. The inflammatory response can be initiated from fat stores [30], or due to uremia, dialysis, or infection [32]. In HD patients, abdominal fat deposition has been linked to inflammation and subsequent increases in mortality risk [33]. In PD patients, visceral fat level was an independent predictor of pulse wave velocity and brachial artery flow–mediated dilatation, and was also one of the risk factors for cardiovascular disease in this population [34]. Our previous study also revealed that larger amounts of adipose tissue were associated with higher serum levels of pro-inflammatory markers and were, therefore, related to the left ventricular diastolic dysfunction [35].

**Figure 3:**
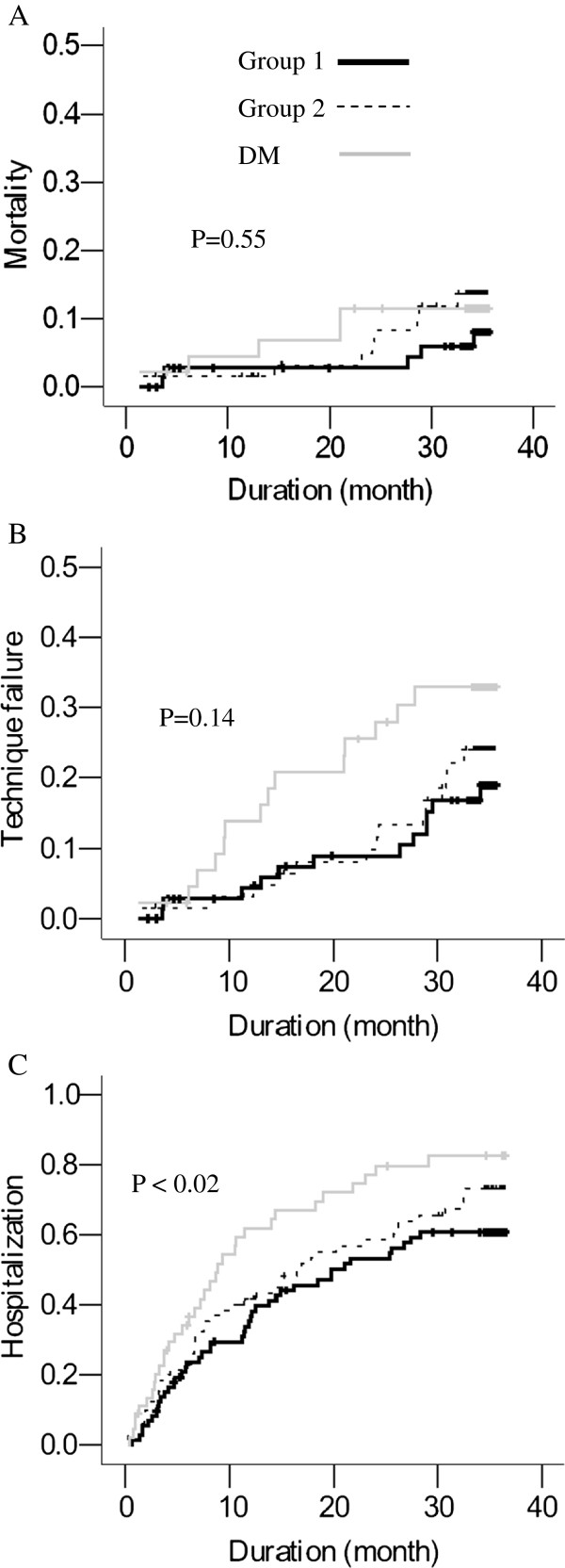
**Kaplan-Meir survival analysis was used to compare the duration of time to mortality (A), technique failure (B), and hospitalization (C) among PD patients.** All patients were categorized into group 1 non-MetS, group 2 MetS and group 3 DM patients. There was no significant difference among these three groups except that DM patients had shorter time to hospitalization.

In our study on PD patients, CRP positively correlated with VFA, SFA, and TFA (Table 2) and remained an independent predictor of fat area after adjusting for sex, age, and BW (Table 3). These results further confirm the relationship between abdominal fat and inflammation in non-DM patients. However, increased abdominal fat and MetS did not influence survival outcomes (Figure 3).

In total, 116 out of 138 (84%) PD patients with hypertension in our study were prescribed medications to control their BP. However, commonly used antihypertensive agents have different effects on insulin sensitivity, which is associated with abdominal fat. Plasma angiotensin is associated with adiposity [36], and RAS blockers can improve glucose homeostasis [37] and reduce abdominal fat [19,20]. Although we conducted a retrospective analysis of patients’ medication use, our PD patients appeared to experience similar RAS blocker–induced effects on VFA. In contrast, traditional beta blockers are known to worsen glucose homeostasis [38]. In the present study, a new generation beta blocker, nebivolol, which may benefit insulin sensitivity [21], was not used. Beta blockers were positively correlated with SFA. Insulin sensitivity might be influenced by these drugs and the abdominal fat accumulation that follows their use, but to date, their clinical significance in PD patients remains unclear.

This statement raises the question of why CRP-associated abdominal fat does not predict survival in PD patients. Our data may explain this phenomenon. First, BMI had a positive correlation with area of abdominal fat; however, the fat area was adjusted for BMI in this study (Table 2). The inverse association between BMI and mortality in dialysis patients has been established [39]. Second, in addition to CRP and age, albumin was positively correlated with VFA (Table 3). Albumin has been shown to be an important predictor of survival in dialysis patients [40]. With respect to the role of MetS, the analysis of the MetS criteria showed that each criterion did not have a uniform effect in PD patients (Table 4). This study population was composed of fewer obese patients and more hypertensive patients. Obesity has been associated with better clinical outcomes in dialysis patients. Another inverse correlation was also noted between blood pressure and CRP levels, with hypertensive patients having lower CRP levels than normotensive patients. Therefore, these multiple associations complicate the effects of MetS and abdominal fat on PD patient survival.

The present study has certain limitations. First, the follow-up period is too short to monitor longer-term outcomes such as CVD events and mortality. Second, the small sample size may have influenced the statistical analysis. Since the overall CVD event rate was low in the present study, more patients should be recruited in future studies to determine the effect of abdominal fat area on the CVD event rate. Finally, related to the survival analysis, all the parameters, including VFA, might vary during the study period. As it will not be feasible to measure VFA at regular intervals with CT scans, this will remain a limitation in future studies on survival outcomes.

In conclusion, both abdominal fat area and MetS are associated with inflammation in PD patients. Although the mechanism of the association remains unclear, the use of RAS blockers and beta blockers are associated with abdominal fat area in PD patients without DM. Abdominal fat area and MetS are associated with BMI and nutritional status, and these had an effect opposite to that of inflammation on patient outcomes. These complex associations decrease the capacity of abdominal fat and MetS to predict PD patient outcomes accurately.

## Competing interests

The authors declare that they have no competing interests.

## Authors’ contributions

J-WH, Y-CL and K-YH carried out the study design, drafted the manuscript and given final approval of the version to be published. C-YY, H-YW, K-LL, C-TS carried out analysis and interpretation of data. C-KW, J-KL, C-KC, H-TC carried out acquisition of data and participated in the sequence alignment. All authors read and approved the final manuscript.
